# A Kriging-Based Approach to Autonomous Experimentation with Applications to X-Ray Scattering

**DOI:** 10.1038/s41598-019-48114-3

**Published:** 2019-08-14

**Authors:** Marcus M. Noack, Kevin G. Yager, Masafumi Fukuto, Gregory S. Doerk, Ruipeng Li, James A. Sethian

**Affiliations:** 1 The Center for Advanced Mathematics for Energy Research Applications (CAMERA), Lawrence Berkeley National Laboratory, Berkeley, CA 94720 USA; 2 Center for Functional Nanomaterials, Brookhaven National Laboratory, Upton, NY 11973 USA; 3 National Synchrotron Light Source II, Brookhaven National Laboratory, Upton, NY 11973 USA; 4 Department of Mathematics, University of California, Berkeley, 94720 USA

**Keywords:** Software, Design, synthesis and processing, Applied mathematics, Computational science, Scientific data

## Abstract

Modern scientific instruments are acquiring data at ever-increasing rates, leading to an exponential increase in the size of data sets. Taking full advantage of these acquisition rates will require corresponding advancements in the speed and efficiency of data analytics and experimental control. A significant step forward would come from automatic decision-making methods that enable scientific instruments to autonomously explore scientific problems—that is, to intelligently explore parameter spaces without human intervention, selecting high-value measurements to perform based on the continually growing experimental data set. Here, we develop such an autonomous decision-making algorithm that is physics-agnostic, generalizable, and operates in an abstract multi-dimensional parameter space. Our approach relies on constructing a surrogate model that fits and interpolates the available experimental data, and is continuously refined as more data is gathered. The distribution and correlation of the data is used to generate a corresponding uncertainty across the surrogate model. By suggesting follow-up measurements in regions of greatest uncertainty, the algorithm maximally increases knowledge with each added measurement. This procedure is applied repeatedly, with the algorithm iteratively reducing model error and thus efficiently sampling the parameter space with each new measurement that it requests. We validate the method using synthetic data, demonstrating that it converges to faithful replica of test functions more rapidly than competing methods, and demonstrate the viability of the approach in an experimental context by using it to direct autonomous small-angle (SAXS) and grazing-incidence small-angle (GISAXS) x-ray scattering experiments.

## Introduction

A core paradigm in experimental materials science is the iterative exploration of the multi-dimensional parameter spaces that underlie materials makeup, synthesis, and processing. In order to establish synthesis-structure relationships and structure-property relationships, scientists iteratively test different material compositions, processing protocols, and environmental conditions. In each ‘loop’ of this process, the scientist analyzes the trends in the available data, and, exploiting their own domain knowledge, selects useful follow-up measurements. The traditional (manual) mode of data collection is costly in the sense that it consumes the attention of a human expert. Moreover, manual experimentation may be inefficient since there is no guarantee that the measurement selection is optimal, especially if the underlying parameter space is vast and high-dimensional. Finally, a purely manual approach lacks a quantitative metric for deciding when to terminate the investigation; it is left to human judgment whether ‘enough’ data has been collected.

This bottleneck is becoming increasingly troublesome, when measured against the rapid advances in instrumentation, data gathering, and analysis tools. Modern scientific instruments feature improved automation (e.g. robotic sample handling), faster data collection (e.g. improved detectors), and faster response (e.g. faster data workup), thereby enabling ever-greater data collection rates. This massive automation allows for more ambitious and complex scientific problems to be investigated, since larger parameter ranges can be studied, these can be sampled more densely, and because the data collection itself is more systematic and reproducible. However, the enormous potential of improved acquisition rates underscore the need for corresponding improvements in the ability of machines to develop and update experimental measurement plans at a speed commensurate to that of the measurement. What is needed are generalizable decision-making algorithms that autonomously drive experiments without human intervention.

**Figure 1:**
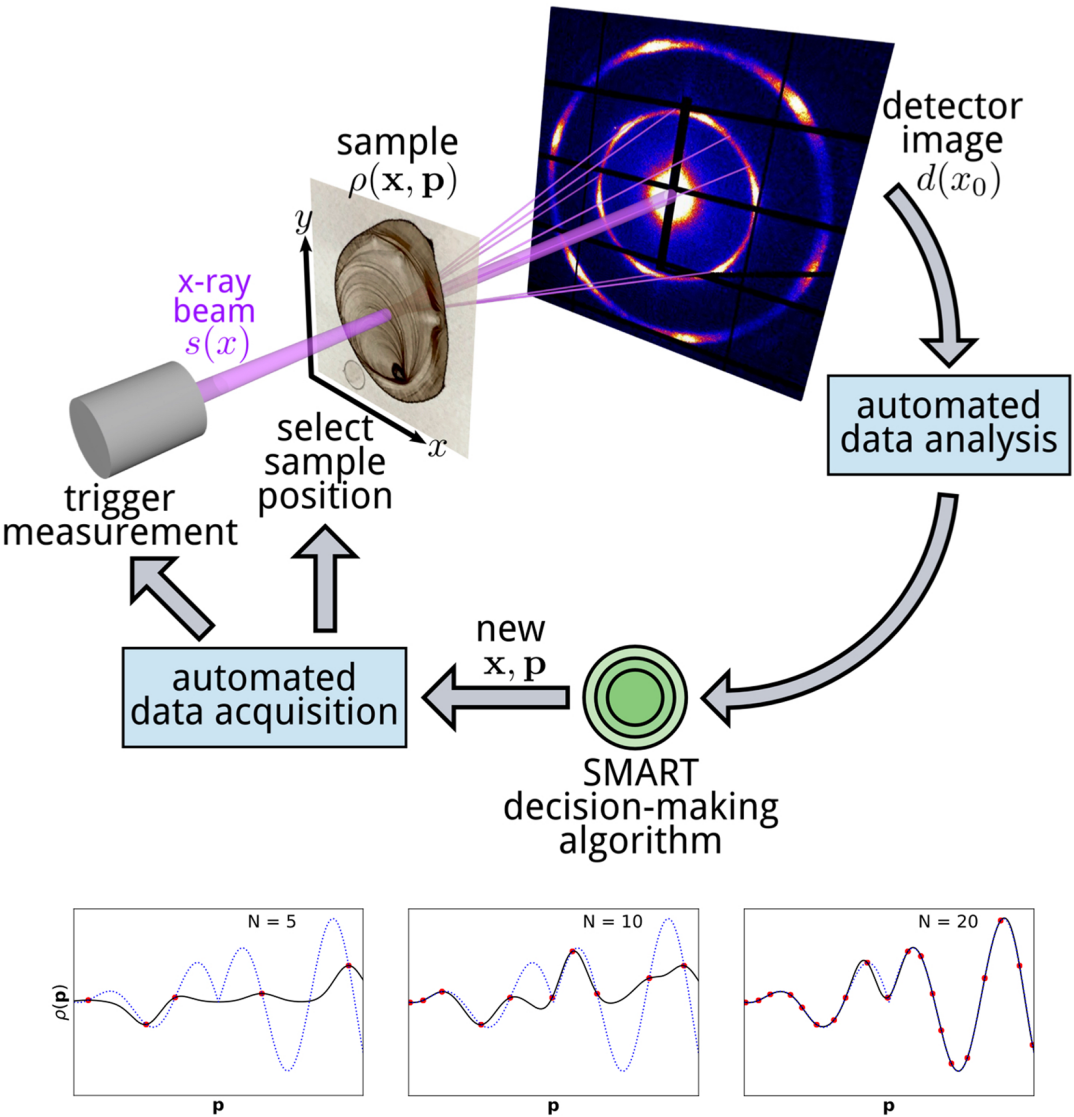
Schematic of an autonomous x-ray scattering experiment. When the measurement is performed, the data acquisition and processing occur automatically. From the processed data, the SMART algorithm selects the next measurement parameters. The graphs (bottom) show an example where a one-dimensional problem is studied. As the number of measurements (*N*) increases, the surrogate model (black) more and more closely matches the actual physical system behavior (dotted blue). Note here that ordinary Kriging does not put emphasis on the characteristic of the function (e.g., high gradients). For this, the surrogate model function has to be invoked explicitly as shown in the results section.

In this work, we develop a general approach for autonomous experimentation that automatically selects measurements from the parameter space that defines a given scientific problem. As a test bed setting, we consider the common experimental case where a class of materials is being studied in order to uncover a set of correlations between measurable properties and a set of controllable synthesis, processing, or environmental parameters (temperature, pressure, etc.). The full landscape of possible materials can be thought of as a multi-dimensional space with a basis, in which each basis vector represents one of the control parameters, and a particular measurement of a particular material is a single point in this space. We refer to the control parameters as ${\bf{p}}$ (position in the parameter space), and the measurement outcomes as $\rho ({\bf{p}})$. The objective in an autonomous experiment is thus to obtain a maximal-quality reconstruction of the true $\rho$ using a minimal number of measurements. As a concrete and illustrative example, we consider this methodology in the context of x-ray scattering experiments. X-ray scattering is a structural measurement technique that consists of shining a bright, collimated beam of x-rays through a material of interest. The resulting far-field scattering pattern is collected on a two-dimensional detector. The features observed in the scattering image (peaks, rings, etc.) encode detailed information about the material’s structure at the molecular and nano-scale. Thus, x-ray scattering allows one to probe material structure or properties for a given sample associated with position ${\bf{p}}$ in the parameter space, where ${\bf{p}}$ includes parameters associated with sample fabrication (composition, annealing time, etc.), as well as those associated with the environmental conditions concurrent with the measurement (temperature, pressure, ambient humidity, etc.) (see Fig. 1). The measurement outcome is a real valued scalar or vector, for instance, the peak intensity of the scattering pattern.

Reconstructing a model for $\rho ({\bf{p}})$ can be thought of as solving an inverse problem in which a parameterized model is fit to experimental measurement data. The fit acts as a ‘surrogate model’ that interpolates between measured data points and provides a prediction of system behavior in unmeasured parts of the parameter space. A naive approach to selecting measurement points is to simply take measurements along a Cartesian grid in this high-dimensional space with an arbitrarily-selected initial spacing. In a one-dimensional example, one could, for example, perform measurements at ten equidistant, discrete temperatures (${T}_{i},i=\{0,1,\ldots ,9\}$). Interpolation would then result in the function $\rho (T)$, which, one would then hope, reasonably matches the true mapping between temperature and structure for the system under study. However, as the number of parameters rises, this procedure becomes increasingly impractical. Searching across even just four parameters poses a major challenge, since, using a similar approach, 10,000 measurements would have to be performed. Another approach is to choose new parameter sets randomly or by intuition. These approaches will successfully sample the space, but can be quite inefficient^1^, since, as the number of performed measurements increases, the probability of choosing non-optimal parameters increases. These approaches can also suffer from experimental bias, and may miss performing valuable but less obvious experiments.

When one seeks to optimize a specific characteristic of a material, a one-variable-at-a-time (OFAT/OVAT) approach^2^ is often employed. For non-convex models, this approach will most likely fail. In the study of modern functional materials, the underlying synthesis/processing parameter spaces tend to be high-dimensional, while material response to these parameters tends to be highly complex (non-monotonic, non-separable processing parameters, etc.). Thus, naive approaches do not fare well. The selection of experiments in high-dimensional spaces has been studied extensively in the context of design-of-experiment (DOE), which can be defined as the application of geometric principles to statistical sampling^3^. Traditionally in these approaches, an experimental plan is formulated at the outset by selecting a geometry that reasonably covers the parameter space. These approaches do not leverage the outcome of past experiments and thus do not provide a way to generate an adaptive measurement scheme. DOE methods can be combined with machine learning techniques to increase efficiency and to deliver a meaningful measure of quality of the final model^2^.

In recent years, several methods have been proposed to enable some form of autonomous experimental decision-making. Most existing approaches are based on machine learning techniques^4–8^, since the main goal is always to find a surrogate model for an unknown, data-based operator, which maps from parameters into measurement results. Scarborough, N. M. *et al*.^5^, for instance, used a supervised learning approach to find the most optimal locations to probe, in order to gain the most information per measurement and at the same time minimize the dose in diffraction-based protein crystal positioning. The machine learns by using a regression function that is computed by an offline training process. Nikolaev, P. *et al*.^9^ employed a combination of the random forest method and a genetic algorithm to steer nano-tube growth. A related class of algorithms comes from the field of image reconstruction, where the overall goal is to minimize the number of measurements needed to recreate an image^6^. These methods all approximate the real structure, behavior, or characteristics of a sample by a surrogate model (explicitly or implicitly), which can be directly or indirectly optimized in order to steer measurements. Lookman, T. *et al*.^10,11^ motivated the collaboration of surrogate models and numerical function optimization for the search for new material properties. Machine-learning methods have also been used to enhance high-throughput characterization of metallic alloy systems based on x-ray diffraction (XRD)^12,13^. Kusne, A. G. *et al*.^12^ used a machine-learning algorithm to implement rapid classification of XRD-derived structure in ternary composition space with enhanced accuracy, facilitating discovery of a novel magnetic phase^12^. Ren, F. *et al*.^13^ combined existing structural database and physicochemical theories with newly performed XRD experiments on combinatorial sample libraries, to iteratively retrain a machine-learning model for glass-forming regions in ternary composition space; this approach, in which the model was used to both improve theoretical parameters and provide basis for selecting combinatorial libraries to be prepared and measured in the next iteration, led to discovery of new metallic glasses^13^.

In this paper, we present a method, SMART (Surrogate Model Autonomous expeRimenT), that creates a surrogate model using the Kriging method^14^, a kind of Gaussian process regression, to steer autonomous x-ray scattering experiments. Kriging is a statistical technique, originating in the geosciences, that can be used to compute a surrogate model that minimizes an estimated error measure between data points. In addition to the surrogate model, Kriging also naturally provides a variance for every input point ${\bf{p}}$, which we refer to as error. The maxima of this error function are reasonable suggestions for where to localize subsequent measurements^15^, since measurements in high-error parts of the parameter space will necessarily decrease the overall surrogate model uncertainty the most, and are thus of high-value from an information-content point of view. For reasons that will become apparent later, we use a genetic algorithm to find the maximum of the error function. The method we present is agnostic to the underlying meaning of the parameters ${\bf{p}}$: any synthesis, processing, or environmental parameter can be included and even combined. Thus, any experiment that can be formulated in terms of an abstract parameter space of possible measurements can be steered with the help of the proposed method, in which the underlying dependency will be captured by the surrogate model. In principle, Kriging can be used to model a parameter space with any dimensionality and for an arbitrary number of experimental data points. In practice, for Gaussian regression tools such as Kriging, the computation time scales with the number of data points and thus implicitly increases when one tackles higher-dimensional problems since one requires more points to meaningfully sample high-dimensional spaces. It is an active research challenge to increase the number of sampling points used in Gaussian regression methods^16^. Despite this problematic scaling, it is worth noting that for many experimental problems the computation time is not limiting. For instance, in x-ray scattering experiments targeting 2–6 parameters, and collecting hundreds or even thousands of data points, the time required to recompute the Gaussian regression is similar to the time to conduct each measurement (seconds to minutes). We demonstrate autonomous experiments using x-ray scattering, while noting that the method is equally applicable to any other measurement technique. SMART is agnostic to the type of experiment since it generally does not use any raw data explicitly and therefore only depends implicitly on it. It instead utilizes specific quantities the analysis software of a given experiment outputs. As long as this output is a set of real numbers, the proposed algorithm can be deployed as described in the paper. The generality of the proposed approach suggests that imaging, including bio-imaging and tomographic reconstruction, could benefit greatly from the proposed method. To the knowledge of the authors, this is the first time that Kriging in potentially high-dimensional spaces has been combined with function optimization to steer x-ray scattering experiments.

The paper is organized as follows. First, we outline the basic theory. Second, the implementation of the method is described in detail. Third, we present validation experiments on synthetic test functions, followed by practical SAXS and GISAXS experiments performed at a synchrotron beamline, thereby demonstrating the power of this autonomous approach.

## Theory

### Preliminaries

The main goal of SMART is to find the function $\rho ({\bf{x}},{\bf{p}})$ as efficiently as possible. We define elements ${\bf{p}}$ of an n-dimensional vector space $P$, which is equipped with the ${L}^{2}$ norm. The set of all elements of $P$ is compact. We refer to $P$ as the parameter space. The parameters ${\bf{p}}$ are the physical variables that define a particular material, such as composition, temperature, and pressure. We use $\rho$ to refer to the corresponding measurement outcome; either the actual measured values at a given position in the parameter space, or the computed expectation throughout the space. Figure 1 shows a schematic of an autonomous experiment. We include explicit reference to sample location ${\bf{x}}$, to emphasize that as far as the mathematical model $\rho ({\bf{x}},{\bf{p}})$ is concerned, the spatial coordinates ${\bf{x}}$ can be treated on equal footing to, or as a subset of, the parameters ${\bf{p}}$. In cases where one is only considering the ${\bf{x}}$ subset of the parameter space, the algorithm is essentially generating an optimized scanning-probe imaging procedure.

### SMART: Kriging to obtain surrogate models and error functions

In SMART, Kriging, an instance of Gaussian process regression, is used to compute an interpolant that inherently minimizes the estimated variance between the data points and also returns a numerical value for the estimated error. Kriging constructs the surrogate model as a linear combination of weights $w({\bf{p}})$ and data points $\rho ({{\bf{p}}}_{i})$, where $i$ is the index of the *i*th measured data point, not the *i*th component of the vector ${\bf{p}}$. Here, we omit the dependency on ${\bf{x}}$ since it is included in ${\bf{p}}$. The surrogate model is defined by

1 $${\rho }_{s}({\bf{p}})=\mathop{\sum }\limits_{i}^{N}\,{w}_{i}({\bf{p}})\,\rho ({{\bf{p}}}_{i}),$$

where ${\rho }_{s}({\bf{p}})$ is the surrogate model that we have constructed, $\rho ({{\bf{p}}}_{i})$ are the measured values of the true physical model $\rho$ at point ${{\bf{p}}}_{i}$, obtained from the previous measurements.

**Figure 2:**
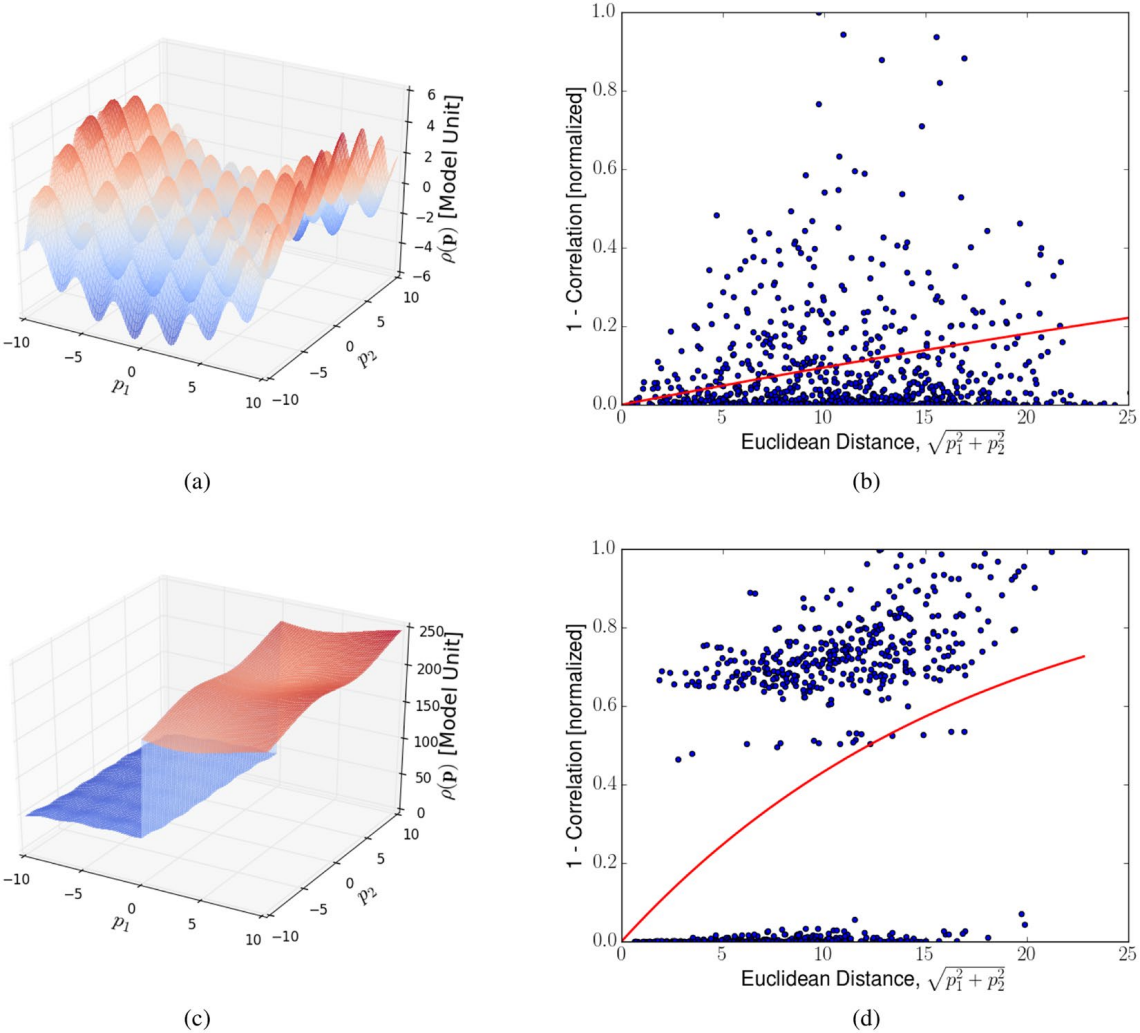
Computed variograms for two different synthetic test functions and randomly chosen measurement locations. The dots represent the difference of data ${\rm{\Delta }}{\rho }_{ij}={(\rho ({{\bf{p}}}_{i})-\rho ({{\bf{p}}}_{j}))}^{2}$. The graph represents the fitted variogram. The variogram encompasses information about correlation between function evaluations at points at different distances and is directly used by Kriging to compute adequate measuring steps. The two test function were chosen to highlight the different behavior of the variogram in different situations; one function, that changes smoothly and the step function with a sudden drop. It is important that the variogram translates the correlation of data correctly, since the true model is never previously known. The variogram is built by fitting a predefined function to the squared difference of existing data points in a least-squares manner and is updated after each performed measurement to account for the most recent data. The squared differences in the data are normalized to unity before the fitting. C and D in equations (4) and (5) are functions of the variogram only. The variogram is a function of the euclidean distance. A low-lying variogram means high correlation of data. A steeply rising variogram means statistical low correlation of data. (**a**) Test function portraying a mathematical model with correlations at many different length scales. (**b**) Variogram, *γ*, for the synthetic test function in (**a**). Model function values are strongly correlated locally, and also over large distances. The average behavior (red line) suggests that local information can be extrapolated to relatively far-away parts of the parameter space. (**c**) Test function portraying a mathematical model with small correlations over large distances. (**d**) Variogram, *γ*, for the synthetic test function in (**c**). While some model function values are highly correlated, there is a large set of pairwise comparisons where the correlation is very poor. The average behavior (red line) suggests that one needs to collect information over relatively short distances in order to confidently reconstruct the model function.

The goal of Kriging is to minimize the mean squared prediction error^14^

2 $${\sigma }^{2}=E[(\rho ({\bf{p}})-\mathop{\sum }\limits_{i}^{N}{w}_{i}({\bf{p}})\rho ({{\bf{p}}}_{i}){)}^{2}]$$

given by

3 $${\sigma }^{2}={C}_{00}-{{\bf{w}}}^{T}\,{\bf{C}}{\bf{w}}-2{{\bf{w}}}^{T}\,{\bf{D}},$$

where the matrices **C** and **D** are defined as

4 $${C}_{ij}=1-\gamma (\parallel {{\bf{p}}}_{i}-{{\bf{p}}}_{j}{\parallel }_{{\rm{2}}}),$$

5 $${D}_{i}=1-\gamma (\parallel {{\bf{p}}}_{0}-{{\bf{p}}}_{i}{\parallel }_{{\rm{2}}}),$$

where ${{\bf{p}}}_{0}$ refers to the position in $P$ where the error is to be estimated and $\gamma$ is the so-called variogram, which is a selectively chosen function that serves as a measure for the correlation of data points depending on the distance between them. In this work, the variogram is defined as

6 $$\gamma =1-{e}^{-ah},$$

where *h* is the euclidean distance between two points $\parallel {{\bf{p}}}_{1}-{{\bf{p}}}_{2}{\parallel }_{{\rm{2}}}$. The variable *a* is chosen in a least squares manner to fit the squared difference of the data. See Fig. 2 for a visualization and more explanation of variograms.

Using minimization of (2) via the Lagrange multiplier method^14^, and omitting the details, we arrive at an expression for the weights

7 $${\bf{w}}={{\bf{C}}}^{-1}({\bf{D}}-\lambda {\bf{1}}),$$

where

8 $$\lambda =\frac{{{\bf{D}}}^{T}{{\bf{C}}}^{-1}{\bf{1}}-1}{{\bf{1}}{{\bf{C}}}^{-1}{\bf{1}}}.$$

Inserting equations (7) and (8) in equation (3) yields the final expression for the error function or the so-called ordinary Kriging variance

9 $${\sigma }^{2}({\bf{p}})={C}_{00}-{\bf{w}}{({\bf{p}})}^{T}{\bf{D}}({\bf{p}})-\lambda ({\bf{p}})$$

It can be seen in equations (7) and (8) that the Kriging weights solely depend on the variogram and the position of the new point ${{\bf{p}}}_{0}$. The matrix inversion of **C** only includes preexisting data points and not the model function value to be estimated at the new point $\rho ({{\bf{p}}}_{0})$. To make the method more sensitive to high gradients of the surrogate model, we can weight the error

10 $${\sigma }_{g}^{2}({\bf{p}})={\sigma }^{2}(1+c|\nabla {\rho }_{s}({\bf{p}}){|}^{2}),$$

where the weight $c\in [0,\infty )$ is a user-defined real number controlling the impact of the gradient on the autonomous experiment. In this work we are choosing this weight ad-hoc. Future work will investigate how this coefficient can be determined autonomously.

### Maximizing the error

**Figure 3:**
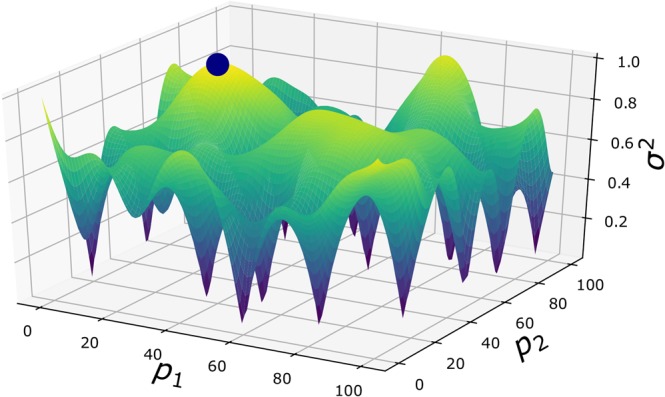
Example of an error function used within the Kriging method. The error function drops to zero at locations where measurements have already been conducted. Measurement errors can be included in the Kriging error, but are commonly neglected for simplicity. If errors are included, the error function will not drop to zero but to the specified variance value of the particular measurement. Between the data points, the function exhibits low curvature ridges and plateaus. In the early stages of autonomous experiments, where there are few data points, maxima are mostly found along the boundary of the domain. These maxima do not generally satisfy the first necessary criterion for optima $(\frac{\partial {\sigma }^{2}}{\partial {\bf{p}}}=0)$. In later stages of an autonomous experiment, maxima will be found inside the domain but curvatures will often be small, which makes derivative-based optimization methods inefficient. Global optimization, however, allows for an efficient optimization for this class of functions over bounded, low-dimensional (<30) sets. In the example shown here, the next measurement will take place at $[20,40]$ (marked).

Equation (9) defines an error function over the parameter space *P*. To implement an autonomous experiment, the SMART algorithm finds the largest error function value and directs the next measurement to be performed at this point. The characteristics of the error function make an efficient optimization difficult. In Fig. 3, we can see that the error function includes a set of narrow ‘pits’ (minima)—which are precisely the locations where measurements have already been taken—with large plateaus and low-curvature ridges in between. The structure of this error function makes conventional steepest-descent/ascent or Newton based optimization difficult. Furthermore, especially in the beginning of the autonomous experiment, the maxima will be located at the boundary of the domain, where optima will not satisfy the first optimality condition (i.e., zero first derivatives). Under these circumstances, a genetic algorithm, is a useful tool to search for the maxima, since it will not be deterred by optima that are on the boundary and do not satisfy $\frac{\partial {\sigma }^{2}({\bf{p}})}{\partial {\bf{p}}}=0$, or by large areas of low curvature in the domain. The greatest drawbacks of genetic algorithms are the inefficiency in high-dimensional spaces, and the uncertainty of finding the global optimum. In our case, these deficiencies are not strongly limiting, since the dimensionality of materials parameter spaces are modest (<30), and finding the global optimum is not strictly necessary, since even a local maximum represents a useful location to perform the next measurement.

### Implementation

The implementation of our decision-making method is divided into four main subroutines or functions. The program execution starts with the *Main* function, which initializes the necessary arrays and calls a while loop which terminates when the surrogate model resembles the target. Resemblance is estimated based on the computed error function; when the error measure drops below a user-defined threshold ${\varepsilon }_{1}$, the experiment is concluded.

**Algorithm 1:**
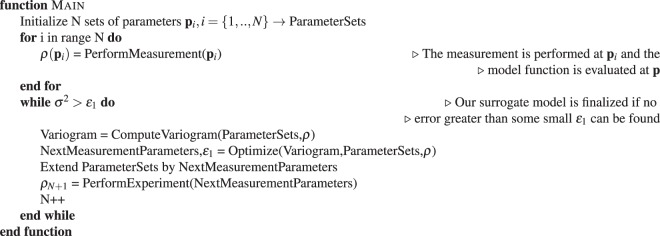
Main Function of SMART.

Within the while-loop in Algorithm 1, the variogram is computed and the optimization is executed, which returns the parameters for the next measurement. The returned parameters represent the point in the parameter space where the genetic optimization algorithm found the greatest estimated error. The computation of the variogram (Algorithm 2) essentially amounts to comparing all existing data points and fitting the differences by a curve (see Fig. 2). The variogram serves as the measure of correlation between the data points.

**Algorithm 2:**
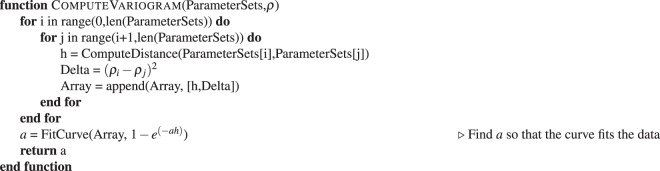
Compute variogram given Data.

The actual Kriging algorithm (Algorithm 4) is called within the *ComputeFitness* function which is called in Algorithm 3. Algorithm 4 computes the variance *σ*^2^ for every individual in a population. The variance serves as fitness in Algorithm 3. The *Optimize* function (Algorithm 3) uses the fitness of each individual to create offspring and eventually to find the maximum of the error function, whose position serves as new parameters for the next measurement in the next iteration.

**Algorithm 3:**
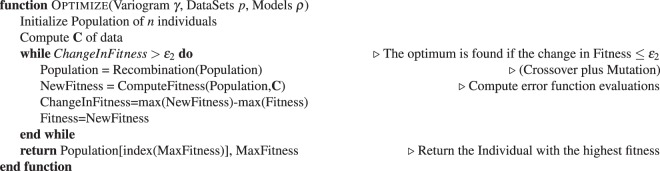
Optimization Function.

**Algorithm 4:**
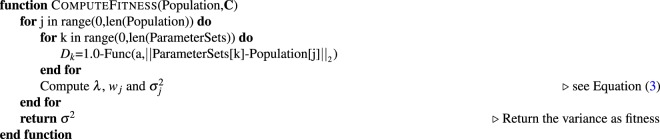
Compute Fitness for all Individuals in Population.

## Tests of the SMART Methodology

In this section we execute two types of tests. First, we compare the SMART method to simpler data-taking strategies: a random search, a uniform-grid-based experiment, and a largest-unexplored-region (LUR) autonomous experiment. To assess the relative quality of measurement strategies, we compare the total prediction error (mismatch between current surrogate model and true $\rho ({\bf{p}})$) vs. number of measurements. Although any measurement strategy should converge to a perfect reconstruction (zero error) after a large number of measurements, better decision-making algorithms will converge to a given error level more rapidly (i.e. using a smaller number of measurements). Second, we challenge the SMART method in exploring several synthetic test functions, each of which presents a particular difficulty. For the results described herein, synthetic autonomous experiments begin with 10 randomly chosen measurements, which is necessary to seed the exploration algorithm. All subsequent parameters are predicted using the proposed method.

### Comparison to grid-based, random-choice and LUR autonomous experiments

**Figure 4:**
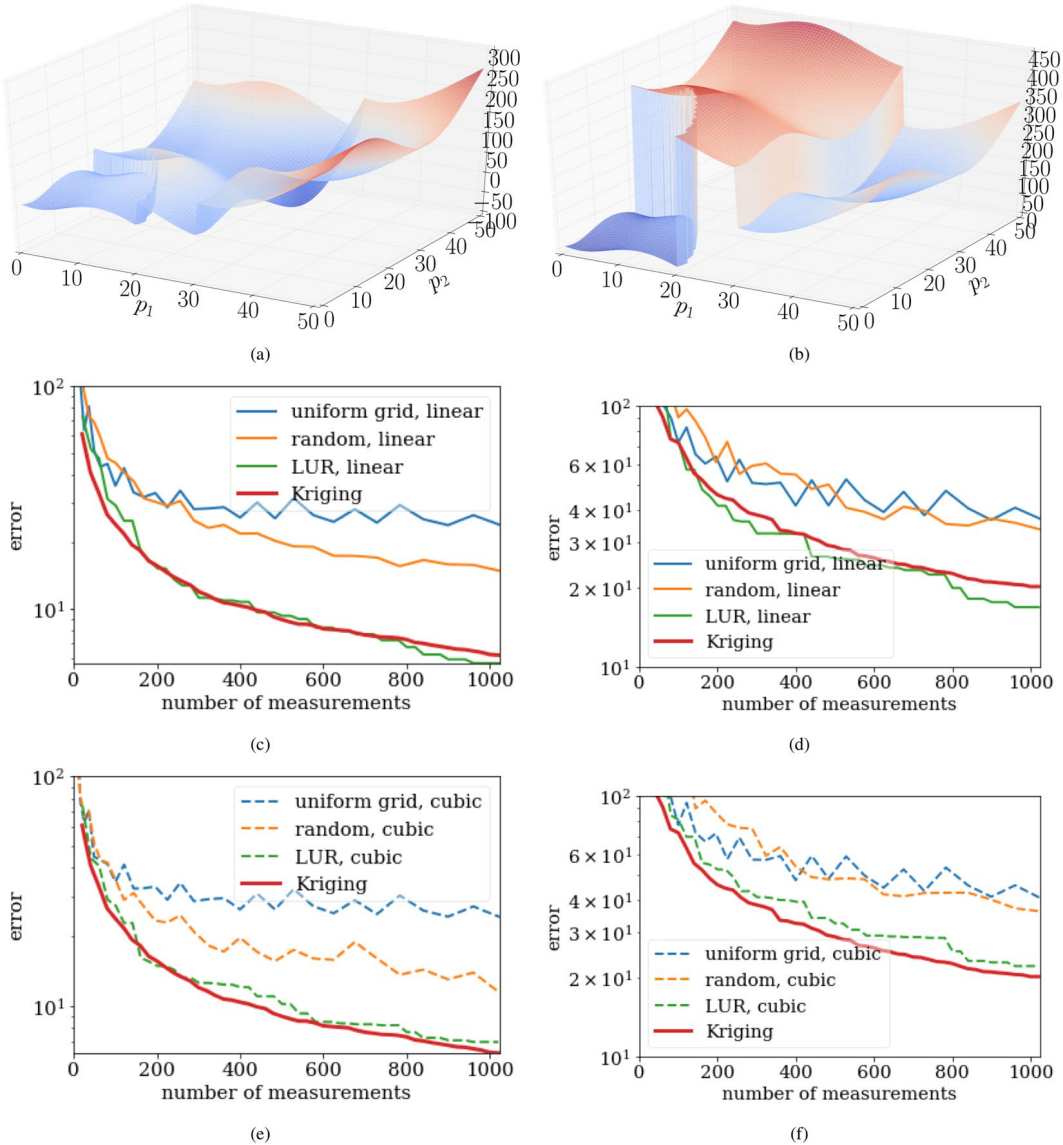
The synthetic test functions defined in equation (12) and (13), and the corresponding error graphs depicting the agreement of the surrogate and mathematical model depending on the number of performed measurements for several different methods. Note that the two synthetic test functions are very similar, but comprise steps of different magnitudes. The difference in magnitude of these steps mainly accounts for the different results seen in the error graphs. A more prominent step will translate into a finer sampling of the space by Kriging. (**a**) The synthetic test functions defined in equation (12). Note the two steps. (**b**) The synthetic test functions defined in equation (13). Note the two large steps. (**c**) Graph depicting the error defined by equation (14) when approximating the synthetic test function (12), shown in (**a**). The data set is interpolated using linear interpolation. (**d**) Graph depicting the error defined by equation (14) when approximating the synthetic test function (13), shown in (**b**). The data set is interpolated using linear interpolation. (**e**) Graph depicting the error defined by equation (14) when approximating the synthetic test function (12), shown in (**a**). The data set is interpolated using cubic interpolation. (**f**) Graph depicting the error defined by equation (14) when approximating the synthetic test function (13), shown in (**b**). The data set is interpolated using cubic interpolation.

In order to compare the performance of different methods, we define a set of synthetic test functions, which are constructed so as to contain both smooth and sharp variations, given that realistic parameter spaces in scientific experiments may contain both continuous variation of parameters, as well as discontinuous jumps (e.g. boundaries in phase diagrams). For simplicity we use a two-dimensional parameter space:

11 $$P=[0,50]\times [0,50]$$

and the following test functions:

12 $$f({{\bf{p}}}_{0},{{\bf{p}}}_{1})=\{\begin{array}{c}8-[2{p}_{0}\,\sin (\sqrt{|{p}_{0}|})+2{p}_{1}\,\sin (\sqrt{|{p}_{1}|})]+140\,{\rm{i}}{\rm{f}}\,{p}_{0}\ge 30\\ 8-[2{p}_{0}\,\sin (\sqrt{|{p}_{0}|})+2{p}_{1}\,\sin (\sqrt{|{p}_{1}|})]+160\,{\rm{i}}{\rm{f}}\,\sqrt{{p}_{0}^{2}+{p}_{1}^{2}}\ge 20\\ 8-[2{p}_{0}\,\sin (\sqrt{|{p}_{0}|})+2{p}_{1}\,\sin (\sqrt{|{p}_{1}|})]\,{\rm{e}}{\rm{l}}{\rm{s}}{\rm{e}}\end{array}$$

and

13 $$f({{\bf{p}}}_{0},{{\bf{p}}}_{1})=\{\begin{array}{c}8-[2{p}_{0}\,\sin (\sqrt{|{p}_{0}|})+2{p}_{1}\,\sin (\sqrt{|{p}_{1}|})]+200\,{\rm{i}}{\rm{f}}\,{p}_{0}\ge 30\\ 8-[2{p}_{0}\,\sin (\sqrt{|{p}_{0}|})+2{p}_{1}\,\sin (\sqrt{|{p}_{1}|})]+360\,{\rm{i}}{\rm{f}}\,\sqrt{{p}_{0}^{2}+{p}_{1}^{2}}\ge 20\\ 8-[2{p}_{0}\,\sin (\sqrt{|{p}_{0}|})+2{p}_{1}\,\sin (\sqrt{|{p}_{1}|})]\,{\rm{e}}{\rm{l}}{\rm{s}}{\rm{e}}\end{array}$$

which are plotted in Fig. 4. The second function (13) intentionally includes large jumps in the function value, as a test of how the various methods handle large discontinuities in the target function value.

For the grid based approach, we divide the domain in a number of squared sub-domains. The measurements in the grid-based approach are performed at the intersections of the sub-domain boundaries. As the name suggests, the random-choice-based method selects the new measurement parameter randomly. The largest-unexplored-region-based approach starts by placing measurements at the corners of the domain *P*. Subsequent measurements are placed in the center of the largest circle that can fit between the existing data points. The procedure continues until the desired number of measurements have been performed. We compare these methods for linear and cubic interpolation by looking at the error defined by

14 $${\rm{error}}=\frac{{\int }_{0}^{50}\,{\int }_{0}^{50}\,|f({\bf{p}})-{\rho }_{s}({\bf{p}})|d{\bf{p}}}{{50}^{2}}.$$

Figure 4 shows the synthetic test functions and the errors as a function of the number of measurements performed. As would be expected, the random-choice strategy performs very poorly. Such a method does not take advantage of the structure of the measured data, nor does it attempt to spread measurement points uniformly throughout the parameter space. The grid approach can be thought of as the most standard experimentalist approach. It intentionally spreads measurements uniformly throughout the parameter space, but does so without any regard to existing data. Surprisingly, this approach performs even worse than random sampling. Both LUR and Kriging peform better. These methods essentially proceed in a ‘coarse-to-fine’ mode, where they initially spread measurements over the entire parameter space (yielding a coarse-grained reconstruction), and then progressively place new measurements in unexplored areas, thereby filling in finer details (yielding a progressivley finer-grained reconstruction). The LUR method is conceptually and computationally simple, but also does not take into account the structure of the measured data. The Kriging method, by contrast, measures (through the variogram) the length-scale over which the data tends to vary. By incorporating this additional information, Kriging is able in most cases to converge slightly faster than LUR.

### Smooth synthetic test function

We consider a synthetic test function in two dimensions that is continuous and also smooth. The function includes both large-scale variations (over the entire space) and more local oscillations and is defined by

15 $$\rho ({\bf{p}})=\,\sin (2{p}_{0})+\,\sin (2{p}_{1})+{(\frac{{p}_{0}}{5})}^{2}-{(\frac{{p}_{1}}{5})}^{2},$$

over

16 $$P=[\,-\,10,10]\times [\,-\,10,10].$$

**Figure 5:**
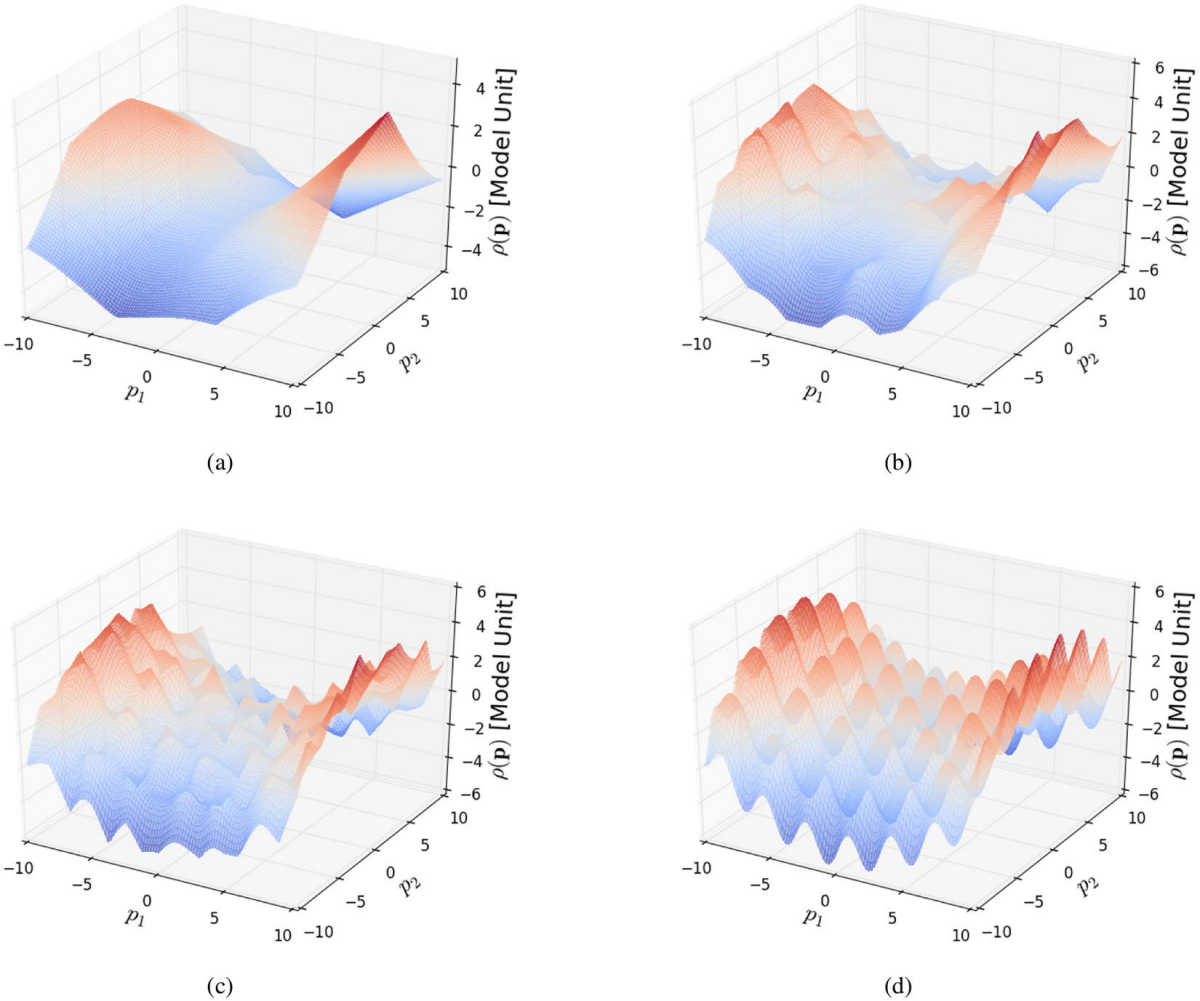
The synthetic test function and the surrogate model. The surrogate model increasingly resembles the synthetic test function. Surrogate model after (**a**) 20 measurements, (**b**) 100 measurements and (**c**) 200 measurements. (**d**) Synthetic test function defined in equation (15).

The results of the progressive SMART-guided sampling of this space can be seen in Fig. 5. The surrogate model begins by yielding a coarse-grained reconstruction of $\rho$, which correctly depicts the overall trend throughout the space even with a small number of measurements (Fig. 5a). As the number of measurements increase, SMART adds data points to progressively reconstruct the more local features. The presence of these local features is quantitatively captured by the global variogram as new measurements probe progressively smaller size-scales. The final reconstruction (Fig. 5c) is a highly-faithful replica of the underlying true function (Fig. 5d).

### Non-differentiable and discontinuous test function

The surrogate model, computed by Kriging using the exponential variogram, is by construction continuous and differentiable, except at point where measurements were performed, as long as the variogram is chosen to have these same characteristics. However, in experimental measurements, certain $\rho$ values may be discontinuous, leading to a non-differentiable function over *P*. For instance, some experimental measures may essentially be binary classifications (e.g. whether the material forms a particular crystallographic structure), or may exhibit very rapid changes over very narrow ranges of **p** (e.g. near phase boundaries). It is thus important to investigate whether the proposed approach handles such cases properly. Therefore, we investigate a synthetic test function that is neither differentiable nor continuous:

17 $$\rho ({\bf{p}})=\{\begin{array}{cc}\frac{25}{17}({p}_{0}-5)+14, & {\rm{f}}{\rm{o}}{\rm{r}}\,{p}_{0} > 21.961143\,{\rm{a}}{\rm{n}}{\rm{d}}\,{p}_{1} > 25\\ \frac{25}{17}({p}_{0}-5)+14, & {\rm{f}}{\rm{o}}{\rm{r}}\,{p}_{0} > 21.961143\,{\rm{a}}{\rm{n}}{\rm{d}}\,{p}_{1}\le 25\\ \frac{-\,25}{18}({p}_{0}-50)+50, & {\rm{f}}{\rm{o}}{\rm{r}}\,{p}_{0}\le 21.961143\,{\rm{a}}{\rm{n}}{\rm{d}}\,{p}_{1} > 25\\ \frac{-\,25}{18}({p}_{0}-50), & {\rm{e}}{\rm{l}}{\rm{s}}{\rm{e}}.\end{array}$$

**Figure 6:**
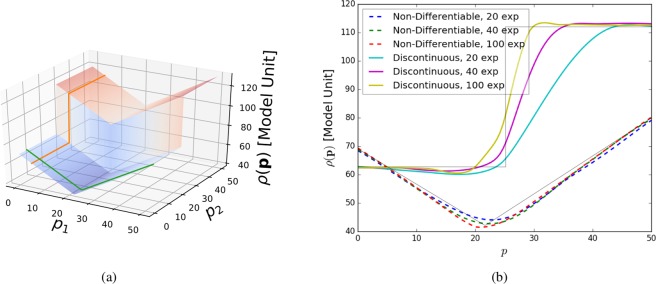
Test of using SMART on a non-differentiable and discontinuous synthetic test function. The surrogate model is able to approximate the underlying non-smooth function as more and more data points are collected. (**a**) Non-differentiable and discontinuous synthetic test functions. The slices, illustrated in (**b**) are indicated. (**b**) One-dimensional slices through the surrogate model showing the behaviour for non-differentiable and discontinuous test functions. Note how the smooth surrogate model approaches the shape of the test function increasingly with increasing number of measurements.

The synthetic test function and the result of the autonomous experiment are shown in Fig. 6. Although the surrogate model is designed to be continuous, it is able to approximate high-gradient areas once data points in those areas are available. As can be seen (Fig. 6), the surrogate model begins to more and more closely match the discontinuous behavior as more measurement points are added. Thus discontinuities and non-differentiability do not by themselves pose a problem. Discontinuous changes in $\rho$ over a space are easily detected by the significant change in measured values. SMART will react to this sudden change by globally using a smaller sampling size. It is worth noting, however, that highly localized discontinuous features in the space being explored—that is, features which do not have any ‘extended footprint’—will be difficult to find using SMART (or any other searching method).

### A three-dimensional physical test function

**Figure 7:**
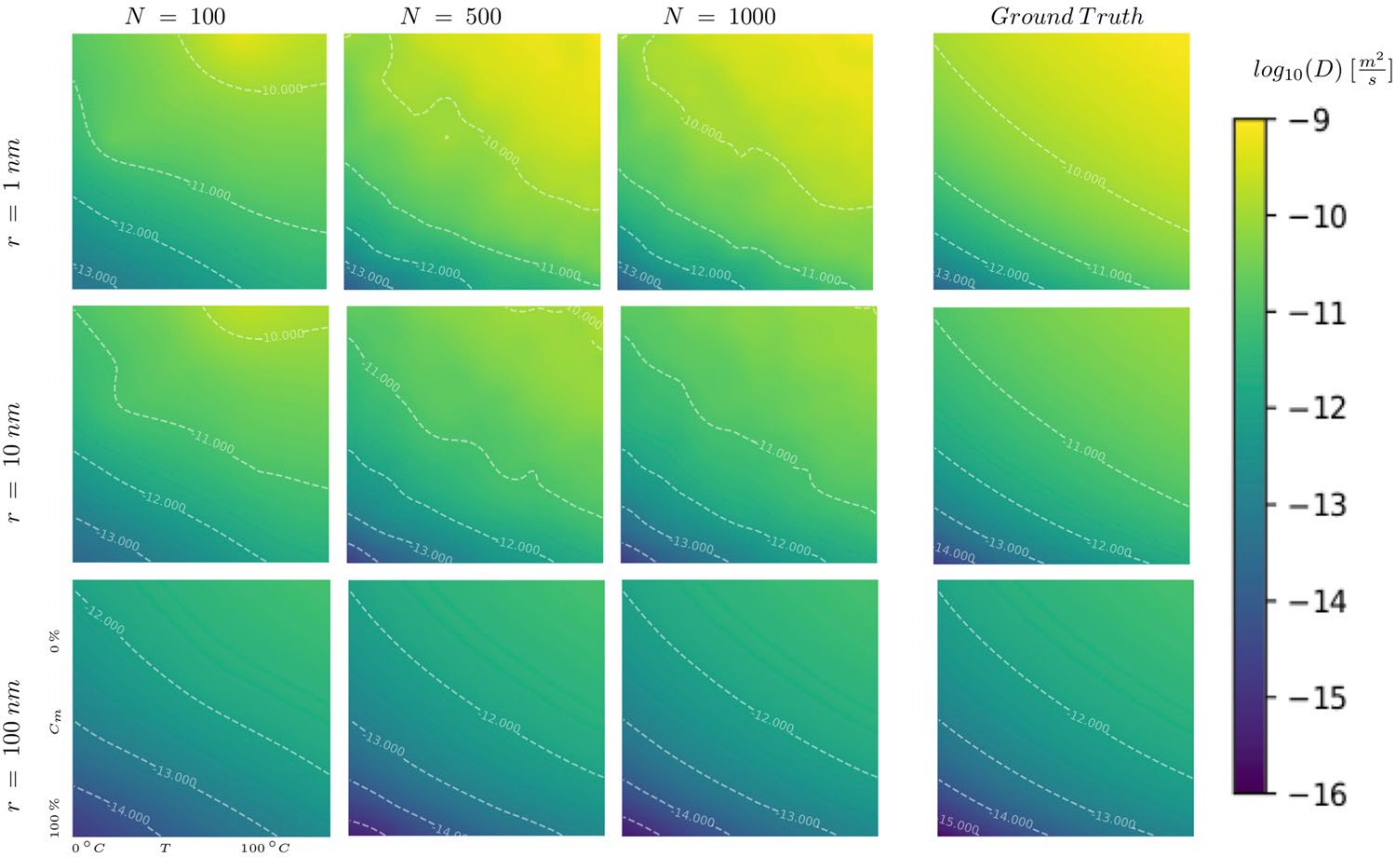
The reconstruction of the physical test function defined in equation (18) after a growing number of steered measurements (*N*). The resemblance becomes more pronounced with an increasing number of measurements.

**Figure 8:**
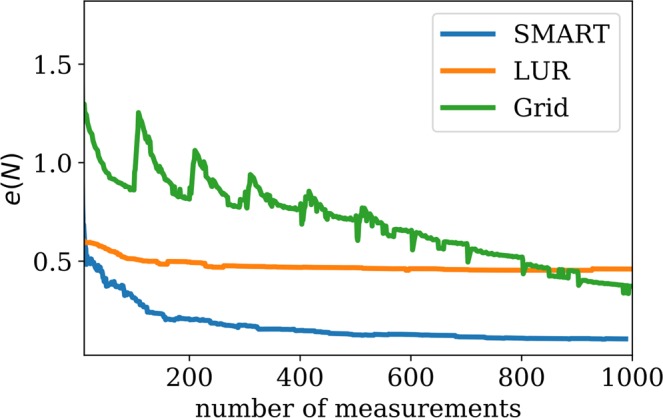
The mean absolute percentage error for the grid-based method, the LUR method and SMART when approximating the physical test function (18). We can see that the SMART method increases its lead as the dimensions of the parameter space increase.

To demonstrate the functionality of the method in more than two dimensions, we show the results for a three-dimensional physical test function. The model to be approximated is the diffusion coefficient $D=D(r,T,{C}_{m})$ for the Brownian motion of nanoparticles in a viscous liquid consisting of a binary mixture of water and glycerol:

18 $$D=\frac{{k}_{B}\,T}{6\pi \mu r},$$

where $r\in [1,100]\,{\rm{n}}{\rm{m}}$ is the nanoparticle radius, *k*_*B*_ is Bolzmann’s constant, $T\in [0,100]\,^\circ {\rm{C}}$ is the temperature and $\mu =\mu (T,{C}_{m})$ is the viscosity as given by Ref.^17^, where ${C}_{m}\in [0.0,100.0] \%$ is the glycerol mass fraction. The diffusion coefficient of nanoparticles in complex fluids can be measured by x-ray photon correlation spectroscopy (XPCS), a coherent x-ray scattering method, which is available at modern x-ray light sources^18,19^. This example emphasizes the need for an autonomously steered experiment. We are putting ourselves in the situation of a beamline user who wishes to determine the behavior of the model that depends on three parameters. We are assuming that, because of time constraints, only a relatively limited number of measurements can be conducted (1000). The options are, either a grid-based method, the LUR method or SMART. For the grid based measurements, each interval was divided into nine equal sub-divisions, bringing the total number of measurements to 1000. The reconstructed model for different radii can be seen in Fig. 7. The error convergence can be seen in Fig. 8. The result shows that, moving towards higher dimensional spaces, the proposed method maintains and even increases its superiority.

## Experimental Validation

In order to validate the concept of autonomous experimentation—and our algorithmic implementation specifically—we carried out a set of experiments at a synchrotron x-ray scattering beamline. Experiments were conducted at the Complex Materials Scattering (CMS, 11-BM) beamline at the National Synchrotron Light Source II (NSLS-II), Brookhaven National Laboratory.

Experimental control was coordinated by having three separate Python-based software processes communicating with each other using a network-shared file system. The beamline was controlled using the *Bluesky* software environment^20^, which enabled control of hardware components (sample positioners, sample exchanger, beam shutter, detector, etc.), triggering of data acquisition, and storage of experimental data and metadata. Data analysis was performed using the *SciAnalysis* software package^21^, which was used to automatically load, reduce, process, and fit experimental data, and thereby generate the experimental $\rho$ signals that are fed into the decision-making algorithm. Experimental decision-making was implemented using our custom-written Python implementation of the SMART algorithm. The SMART code analyzes all available experimental data and issues a request for the next sample position to measure, which is then read and executed by the beamline control software. These three software processes were launched in parallel and allowed to proceed without human intervention for the duration of the experiment.

For the experiments discussed below, the x-ray beam was set to a wavelength of 0.918 Å (13.5 keV x-ray energy) using a multilayer monochromator, and small-angle x-ray scattering (SAXS) patterns were recorded on a photon-counting area detector (DECTRIS Pilatus 2M) located downstream of the sample. For the experiments on block-copolymer thin films, grazing-incidence SAXS (GISAXS) measurements were carried out with a beam size of 0.2 mm wide × 0.05 mm high, an incident angle of 0.12°, and a sample-to-detector distance of 5 m. For the experiments on a nanoparticle coating, transmission SAXS measurements were performed with a beam size of 0.2 mm × 0.2 mm and a sample-to-detector distance of 3 m.

### Exploration of multi-dimensional material assembly

**Figure 9:**
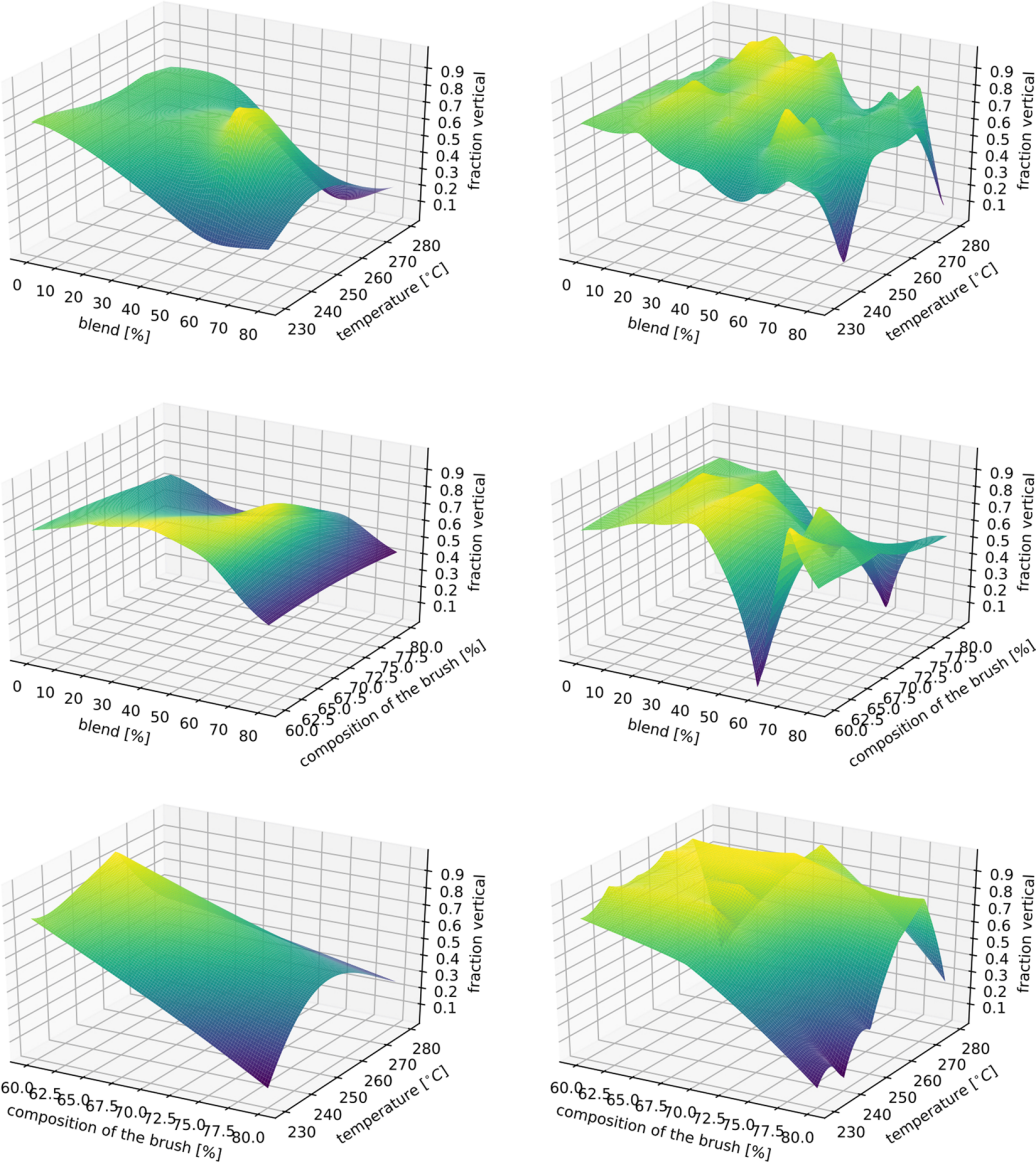
The reconstruction of the model function after 20 (left) and 96 (right) steps for all combinations of the parameters. SMART can provide a decent notion of the model very early on in the experimental process, while, for instance, a grid-based approach will deliver an incomplete picture if not finished. Therefore, the experiment can be conducted using fewer samples.

For this experiment, GISAXS measurements were carried out to probe the morphology of nanostructures formed by block-copolymer thin films on silicon substrates. Block-copolymers are a class of self-assembling polymeric materials–that is, they spontaneously form well-defined nanostructures when the material is heated^22^. The structure that forms can be measured using SAXS, and depends on both material properties (composition, additives, substrate type, etc.) and annealing protocol (temperature, time, etc.). For instance, ordered domain size can be quantified by the inverse of the SAXS peak width^23^. Recent studies have demonstrated how one can control the ordering in these materials by blending; in particular, by mixing block-copolymer materials together with short-chain polymer additives. This noticeably improves the quality of ordering^24,25^, but the resulting materials science problem is multi-dimensional (ternary blend composition, film thickness, substrate surface energy, annealing temperature, annealing time) and thus challenging to study. Here, we demonstrate how autonomous measurements can assist in exploring the underlying multi-dimensional parameter space. For the results presented here, a set of thin film samples of polystyrene-*block*-poly(methyl methacrylate) (PS-*b*-PMMA) were fabricated varying three parameters. Firstly, the blend composition (mass fraction of homopolymer PS and PMMA additives), which was varied from 0% (pure block-copolymer) to 80% (heavily plasticized). Secondly, the composition of the brush applied to the substrate (denoted by the percentage of PS relative to PMMA), which controls substrate surface chemistry, was varied from 60% (roughly equal interactions with PS and PMMA) to 80% (preferential to PS). Finally, the film annealing temperature (*T*) was varied from 230 °C to 280 °C. From the x-ray scattering patterns, we computed the ‘fraction vertical’ (amount of nanostructures oriented vertically vs. horizontally) by analyzing the relative intensity of two distinct scattering peaks. Samples were placed into a queue at the beamline, and a sample-exchange robot was used to load a target sample into the measurement position, as directed by the SMART algorithm. An important implementation detail is that the available samples do not continuously cover the underlying parameter space; instead, the available samples represent a discrete set of points within the space. This common experimental constraint would seem–at first glance–to be incompatible with the SMART algorithm, since it naturally operates on continuous multi-dimensional spaces. Nevertheless, we found that SMART can easily be adapted to these kinds of experimental problems by simply ‘snapping’ the measurement suggestions of the algorithm onto the nearest available sample. Snapping works by projecting the actual suggestion to closest allowed location. As can be seen in Fig. 9, application of SMART to this experimental problem allows the general structure of the parameter space to be reconstructed using a modest number of measurements. Overall, this experimental example demonstrates that SMART can be relatively easily applied to handle problems of experimental interest. In particular, it can be used to explore multi-dimensional parameter spaces defined by materials parameters, and can be used in cases where exploring of parameters must be limited to discrete values.

### Two-dimensional SAXS imaging of a nanoparticle coating

**Figure 10:**
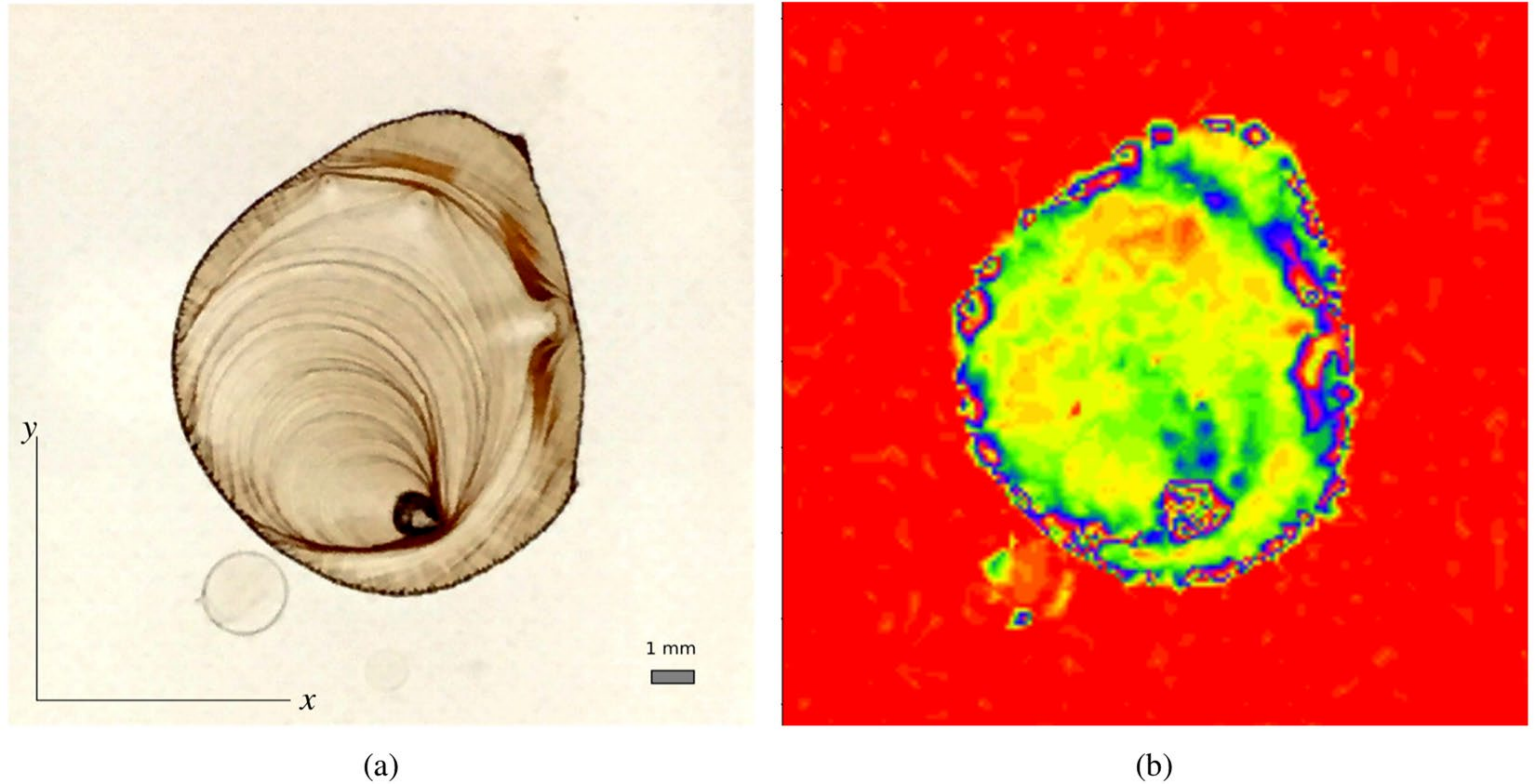
The SMART decision-making algorithm was used to drive an autonomous imaging experiment, wherein a nanoparticle coating was measured by scanning an x-ray beam over the sample surface. (**a**) Optical image of the sample used for the autonomous imaging experiment. The sample consists of a “coffee-ring” pattern that arises from evaporation of a nanoparticle-containing solution. The dark stripes are regions of significant nanoparticle deposition. In these regions, nanoparticles are locally (at the nanoscale) packed into arrays. This optical image can be compared to the reconstruction shown in Fig. 11. (**b**) The final reconstruction (surrogate model) obtained by using all the SAXS peak intensity data acquired during the experiment, taken at more than 4,000 distinct $(x,y)$ positions. The x-ray beam illumination area for each individual measurement was $0.2\,{\rm{mm}}\times 0.2\,{\rm{mm}}$ in size.

In this experimental test, we used SMART to efficiently map out the two-dimensional structure of a material, as shown in Fig. 10, without any human intervention. The sample consisted of nanoparticle coating on a microscope cover glass created by evaporating a droplet of a toluene solution of 12 nm diameter iron oxide nanoparticles. The drying front creates a characteristic “coffee-ring” pattern—a succession of rings of dense nanoparticle deposition. The sample was probed in a scanning-imaging mode, where at each sample position a transmission SAXS image was collected at normal incidence. The darker regions in the optical image (Fig. 10) are those with high nanoparticle concentration, which leads to nanoparticles self-organizing into superlattices; SAXS measurements at these locations gave rise to strong structure-factor peaks due to the particle packing distance, with the six-fold packing symmetry being frequently apparent (Fig. 1). Regions with little or no nanoparticle coverage exhibited no such peaks and only diffuse background scattering. We quantified the amount of nanoparticle signal within a given SAXS image by generating the one-dimensional (circular average) scattering pattern, and fitting the first-order peak to obtain a measure of scattering intensity.

During the experiment, the SMART algorithm sequentially selects the next point $(x,y)$ for SAXS measurement, so as to image the coffee-ring pattern as efficiently as possible. For comparison purposes, we perform the experiment also using a simple grid approach, as well as the largest-unexplored-region method. To increase sensitivity of the SMART method to edges in the image (i.e., large local gradients), we also test a variant of SMART wherein the error function was weighted by the gradient of the surrogate model. To differentiate these methods, we refer to the fundamental Kriging method as kSMART, and the gradient-enhanced version as gSMART. kSMART needs only a definition of the parameter space limits in order to run successfully and autonomously. gSMART requires the selection of a weighting factor which controls the relative contribution of the Kriging error signal and the surrogage model gradient signal. The role of this weighting factor, as well as how it can be selected autonomously, will be discussed in future work.

**Figure 11:**
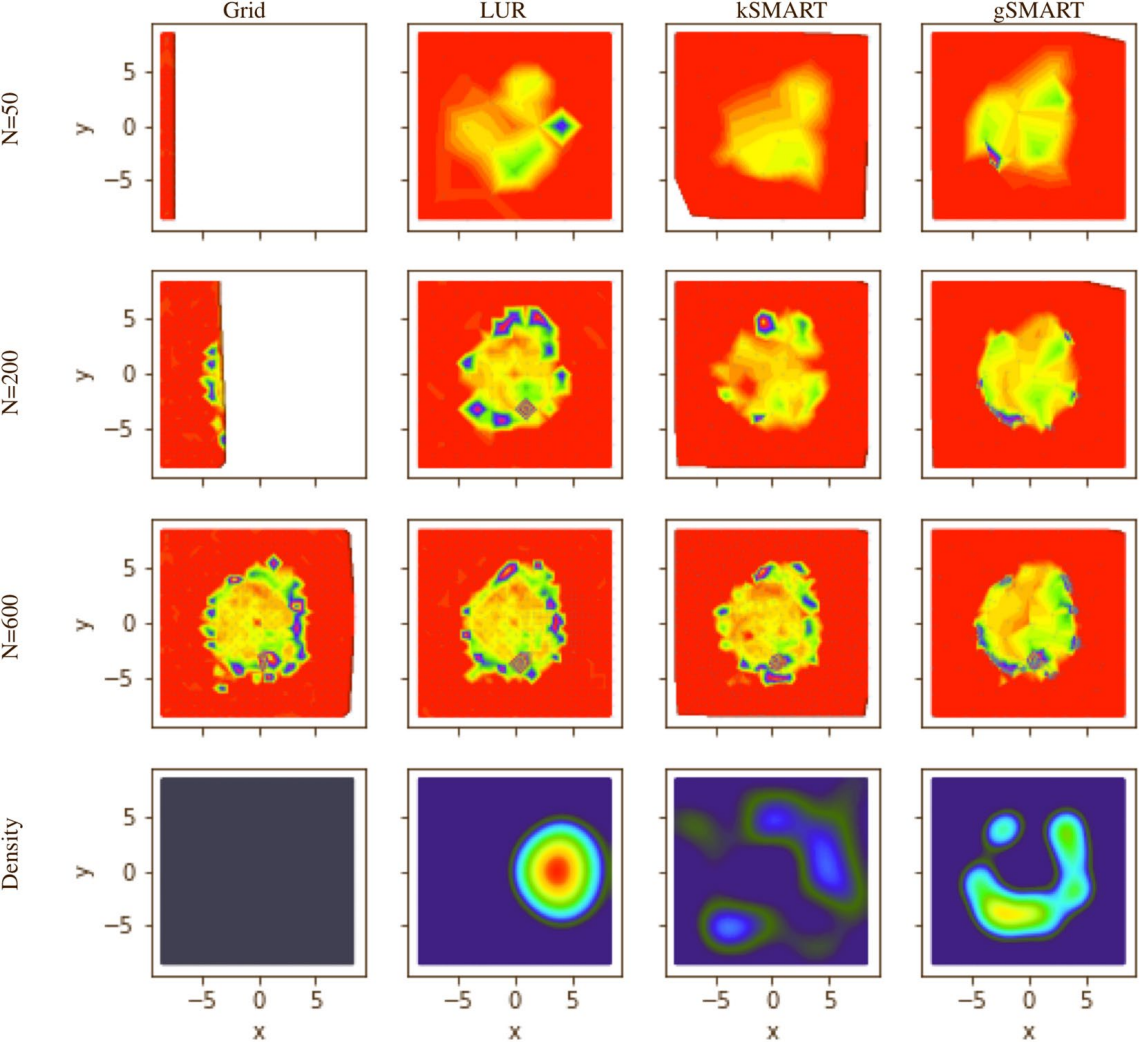
Reconstructed images (surrogate model) from autonomous x-ray scattering imaging experiments. Different decision-making methods were considered: a naive grid scan, selecting the largest unexplored region, the proposed SMART algorithm which measures wherever uncertainty is highest, and a gradient-enhanced variant that localizes measurements near edges in the image. The last row presents a plot of the data density. The error function for gSMART was weighted by ${\sigma }_{g}^{2}={\sigma }_{0}^{2}(1+20|\nabla {\rho }_{s}({\bf{p}}){|}^{2})$ (see equation (10)). The exact locations of the measurements are indicated as points. Note, that the density of measurements is very high around high-gradient regions for the gSMART method. The LUR measurement density is not uniform but unrelated to the underlying model function.

**Figure 12:**
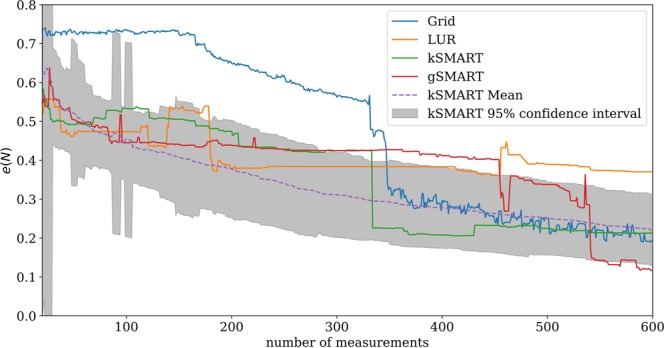
The dependency of the error, defined by $e(N)={\int }_{P}\,{({\rho }_{s}(N)-{\rho }_{s}^{0})}^{2}d{\bf{p}}$, with respect to the ground truth, ${\rho }_{s}^{0}$, created by using all available data for the sample, on the number of measurements *N* for different methods. The error for the grid method drops suddenly when the grid-scan has finished sampling the coffee-ring pattern; thus the location of this drop is particular to the exact sample measured. LUR reduces the error more quickly with a small number of measurements, but thereafter plateaus since it does not take advantage of the structure of the data. The SMART methods perform best, as they first provide a coarse image (rapid initial drop in error) and the progressively refined this image by localizing measurements where they can have the most impact (progressive decrease in error). The figure also contains the result of 100 synthetic experiments, run with the data obtained at the beamline, to show that the proposed method is consistently outperforming the traditional methods. The dashed line shows the mean and the grey area shows the 95% confidence interval.

For this two-dimensional imaging experiment, the parameter space corresponded to spatial coordinates $(x,y)\in P=[\,-\,8.5,8.5]\times [\,-\,8.5,8.5]\,{\rm{mm}}$. The intensity of the first-order scattering peak (obtained via Gaussian fit with a linear background) served as the experimental $\rho$ value. Ten random measurements were performed to ‘seed’ the algorithm—that is, to provide it with an initial set of measurements from which the surrogate and error functions can be computed. The results for the different decision-making approaches are shown in Figs. 11 and 12. The grid approach is simple but not satisfactory. The grid spacing was selected arbitrarily by the human experimenter and is thus certainly not optimal. This measurement strategy also provides only a partial view of the data (at a fixed resolution) until the scan is complete. The LUR algorithm improves upon this by instead providing an incremental reconstruction—that is, it begins with a coarse-grained image of the space and progressively increases resolution by adding points between existing measurements. However, this approach does not take advantage of any features in the measured data and thus misses the opportunity to adapt the data-taking strategy to the structure of the data. The kSMART algorithm also proceeds in a coarse-to-fine mode, generating progressively refined reconstructions as data-taking proceeds. In this case, however, the measurements are distributed so as to most rapidly reduce the error of the surrogate model. As can be seen in Fig. 11, this leads to both complete coverage of the parameter space, but also a slightly non-uniform distribution of the data-taking. That is, SMART has localized measurements where they will add higher value. The gradient-enhanced version (gSMART) emphasizes placing new measurements at the location where there is high variation in $\rho$ (high gradient). This has the effect of localizing data around edges in the image, and thus gSMART achieves high-resolution reconstruction of edges more rapidly than any of the other methods. Overall, SMART thus outperforms the simpler algorithms, both by selecting measurement points in a more rigorous and valuable manner, as well as providing auxiliary information to the experimenter in the form of the surrogate model and the error function.

## Discussion and Conclusion

In this paper, a method to guide autonomous experiments, called SMART (Surrogate Model Autonomous expeRimenT) was presented. SMART exploits the Kriging methodology to construct a surrogate model and an error function, based on the available experimental data. A genetic algorithm is then used to explore the error function, seeking the location with the maximum error. By suggesting this location for the next measurement, the SMART algorithm guides the experiment to maximally reduce uncertainty with each new measurement. Naturally, this leads to a more rapid decrease in the mismatch between the surrogate approximant and the true structure of the data, as compared to more naive data-taking strategies. Through a series of tests on synthetic data as well as during real-world use in an autonomous experiment, we find that the SMART methodology is indeed able to rapidly converge to a faithful replica of the true structure of the data. Moreover, the surrogate and error models are themselves useful to experimenters. The surrogate model acts as a robust fit and interpolation of the experimental data. Kriging yields a non-linear interpolation and extrapolation of existing data, where the shape of the model is determined by computing the average variability in data across length-scales (via the variogram). The surrogate model thus gives the experimenter a useful view of existing data and prediction for the overall trend across the parameter space *P*. The corresponding error function is useful to the experimenter to understand the limitations of the surrogate model, and to assess where further data collection would be valuable. The variogram provides the experimenter with another way to assess their data, by explicitly monitoring variance as a function of distance in the parameter space.

Despite the robustness of the SMART method, there are several trade-offs that should be considered. Our tests have shown that the kSMART (based on Kriging only) method yields a more accurate model approximation than competing methods, at a given number of performed measurements. However, the largest-unexplored-region (LUR) method can sometimes achieve similar performance (see Fig. 4). Kriging uses a variogram, which encompasses two sets of information, the distance between data points and the global variance of data values at those points. From this variogram, Kriging can determine an ‘appropriate’ distance between measurements; that is, one can use the variogram to estimate how closely data-points need to be spaced to robustly sample the space. The LUR method, by contrast, ignores the structure of the data, and instead continually decreases the average distance between measurements as data taking proceeds. This offers no well-defined termination condition. Kriging can be used not only to sample the parameter space in a meaningful manner, but also as a way to terminate data collection (and move on to another task) when a given quality has been reached. The method keeps track of the maximum estimated error across the domain, as a byproduct of the optimization step, which can directly be used to decide whether an experiment can be terminated. In the context of autonomous experimentation, the additional information provided by the variogram and error function can be very valuable in guiding data-taking intelligently. Both methods will eventually achieve a similar average distance between measurements and the accuracy of the model will then be similar as well. Looking at Fig. 12, one could ask why the differences in error are not more severe. The superiority of SMART depends on the characteristics of the model function and the space *P*. If the space *P* is large and high-dimensional, randomly chosen points will show only slightly worse error convergence because a newly chosen point has a high probability of being far away from any other point. As more points are collected, random choice will perform worse since unnecessary, overlapping information is collected. The LUR method will also perform comparably well except that the step length does not depend on the model function which creates slight inefficiencies. In general, one has to keep in mind that all existing methods are sampling methods which need large amount of points to accurately approximate a function. SMART determines a sampling distance based on the global variation of the data. While this ensures correctness, it does not guarantee significant gains compared to the LUR or random method, depending on the unknown model function and the total number of measurements. By simulating the experiment 100 times, we have shown, however, that SMART performs consistently better than the competition (Fig. 12). Other experiments showed a larger difference in error (Fig. 8).

The SMART methodology is also easily adapted to other tasks. For instance, we demonstrated how one can very simply make the data-taking more ‘edge-seeking’ by adding a gradient term to the error function. More generally, the error estimate in Kriging can be augmented with any auxiliary signal in order to enforce different measurement tasks appropriate for different kinds of scientific exploration.

Although the Kriging surrogate model is, by definition of our variogram, continuous and differentiable (except at points where measurements have been performed), the proposed SMART method can be applied to problems where the measurement values change non-smoothly or even discontinuously. The surrogate model is able to approximate such regions in the parameter space using high-gradients. Overall this interpolation can be arbitrarily close to the measurement reality and is more than sufficient for robust guidance in terms of selecting follow-up experiments. Moreover, we note that in practice experimental error and instrumental resolution mean that even a strictly discontinuous change in a value will frequently be recorded as a continuous change. While in this work the measurement error was assumed to be zero and therefore excluded, it will be explicitly included in future work and versions of the code. In case of measurement errors, the interpolant changes from exact to inexact, meaning that the surrogate model does not have to pass though the data exactly. The measurement error in this experiment was negligible compared to the return value, justifying its approximation as zero.

We have demonstrated the use of the SMART algorithm to automatically control real-world x-ray scattering experiments at a synchrotron beamline in two and three dimensions. SMART was able to efficiently image the real-space structure or model function of the sample being probed, by objectively mapping the entire available space (sample area) based on the geometry of the data and the estimated global information content of the predicted surrogate model. Compared to the most commonly used experimental mapping strategy—the uniform-grid-based method—SMART obtained a similar model accuracy about six times faster. However, this real-world test also uncovered several shortcomings. Kriging, as an instance of Gaussian process regression, in its original and ordinary form, is, unable to use any *local* information to search the parameter space more efficiently. It uses a variogram, which, by construction, is a global average that does not keep track of any local information. It is merely a statistical tool to generally express how data points at given distances apart are correlated. This traditional form of Kriging does not put emphasis on local structures that are discovered. However, as we demonstrated in the case of gSMART, Kriging can be adapted to take advantage of additional information. Indeed the addition of gradient information already yields some local information about the structure of the data. The density plots in Fig. 11 clearly show how the gradient-weighted Kriging is more sensitive to interfaces or internal boundaries in the data. This variant of Kriging necessarily includes an additional parameter that controls the relative weight of this gradient term compared to the conventional error term. Determining how to set this weight to achieve optimal results of course depends on the experimental problem, and can leverage the user’s prior knowledge. In future work, we will investigate ways to compute this weight without user input, yielding a fully-autonomous algorithm that considers both global and local variation of the data.

Another weakness of Kriging is the sensitivity to different scales of the parameters, since a simple euclidean norm is used as distance measurement. When the different axes (**p** components) are equivalent (e.g. different spatial directions), the euclidean norm is a fair distance measure. However, when the different axes represent wildly different physical parameters (e.g. temperature and pressure), then this distance measure is not physically meaningful. This problem can be somewhat addressed by normalizing distances along the different axes by the corresponding domain size (of *P*); i.e. scaling the overall parameter space to unity in all directions. However, such a re-scaling is dangerous because naturally different scales will be ignored. The best way to avoid the issue is to choose units so that the parameter limits are of the same order of magnitude. In future work, the issue will be avoided by using anisotropic variograms, which will have different length scales in different directions.

In this work, we used a genetic algorithm to find the maximum of the error function. Although this optimization method performs reasonably well for the kinds of parameter spaces we are considering, a genetic algorithm is not necessarily the ideal choice. In particular, this method loses efficiency as the dimensionality of the parameter space increases. As the data acquisition rate and the dimensionality of the parameter space increase, a genetic algorithm will not keep up with the demands of delivering an optimum in time for the next measurement. With some care and restrictions on potential error functions, other optimization methods could be tested, with some likely exceeding the performance of the genetic algorithm.

In future work, different types of variograms could also be tested. In this work, we used an exponential variogram, which allows for non-differentiability at the data point, is simple and generally applicable. Other variograms, for example the squared exponential kernel or the Matérn variogram class can lead to slightly different results, not impacting the core functionality of the proposed method. In future work, the user will be able to chose from a variety of different kinds of variograms. See Ref.^26^ for an overview of commonly used variograms.

One valuable approach to study may be a meta-model approach in which the algorithm learns based on the greatest error found by Kriging when, and how strongly, to weight the local errors using the gradient of the surrogate model. One great strength of Kriging is the error estimation, which could be used to gradually give the gradient of the surrogate model (or its values themselves, or its curvature) more weight as the Kriging error declines. More generally, there is ample opportunity to refine the SMART methodology by simply adjusting the error model so as to direct the experimentation in a desired manner.

In conclusion, the presented Kriging-based SMART algorithm is a powerful method for selecting candidate measurement points in an experimental context, and thus as a decision-making module in an autonomous experiment context. SMART is able to quickly obtain a grasp of the general shape of the parameter space being probed, in particular when very little is known, a priori, about it. As information is gathered, the shape of the, now partly known, data model can be used to make the search more sensitive to certain features, and thus more efficient.
